# Interactions of Pleiotrophin with a Structurally Defined Heparin Hexasaccharide

**DOI:** 10.3390/biom12010050

**Published:** 2021-12-30

**Authors:** Eathen O. Ryan, Zhoumai Jiang, Hoa Nguyen, Xu Wang

**Affiliations:** The School of Molecular Sciences, Arizona State University, Tempe, AZ 85281, USA; eoryan@asu.edu (E.O.R.); zjiang45@asu.edu (Z.J.); htnguy49@asu.edu (H.N.)

**Keywords:** NMR, glycosaminoglycan, pleiotrophin, cytokine, GAG-binding protein, heparin

## Abstract

Pleiotrophin (PTN) is a potent cytokine that plays an important role in neural generation, angiogenesis, inflammation, and cancers. Its interactions with the polysaccharide glycosaminoglycan (GAG) are crucial to PTN’s biological activities. In this study, we investigated the interaction of selectively protonated PTN with the heparin hexasaccharide ΔUA2S-(GlcNS6S-IdoA2S)_2_-GlcNS6S using solution NMR. The use of a structurally defined oligosaccharide and selectively protonated PTN enabled us to obtain intermolecular contacts using unfiltered NOESY experiments, significantly increasing the amount of high-resolution structural information obtainable. Our data showed that PTN’s arginines, lysines, and tryptophans in the two structured domains have strong interactions with the 2-O-sulfated uronate protons in the heparin hexasaccharide. Consistent with the NMR data is the observation that 2-O-desulfation and N-desulfation/N-acetylation significantly decreased heparin hexasaccharides’ affinity for PTN, while 6-O-desulfation only modestly affected the interactions with PTN. These results allowed us to hypothesize that PTN has a preference for sulfate clusters centered on the GlcNS6S-IdoA2S disaccharide. Using these data and the fact that PTN domains mostly bind heparin hexasaccharides independently, models of the PTN-heparin complex were constructed.

## 1. Introduction

Pleiotrophin (PTN) is a powerful mitogenic and angiogenic cytokine associated with many cellular processes. In particular, PTN plays an important role in early CNS development and stimulates neurite growth in spinal cord injuries [1,2,3]. It also exerts a protective effect on addiction-associated neurotoxicity and is known to modulate the activities of microglial cells [4,5,6,7,8,9,10]. Abnormal levels of PTN have been associated with a number of diseases, including many types of cancer and inflammatory diseases [11,12,13,14,15,16,17,18,19,20]. Recently, it has also been connected with adipocyte differentiation and obesity [21,22,23,24]. The source of PTN’s pleiotropy is most likely connected with PTN’s ability to bind multiple receptors. One class of receptors that play an especially prominent role in PTN signaling is proteoglycans. Proteoglycans are glycoproteins that carry one or more chains of polysaccharides belonging to the glycosaminoglycan (GAG) family. GAGs are a diverse group of linear polysaccharides composed mainly of disaccharide repeating units bearing different degrees of sulfation. So far, PTN’s interactions with the chondroitin sulfate (CS) proteoglycan receptor-type protein phosphatase ζ (PTPRZ/phosphacan) and heparan sulfate (HS) proteoglycans such as syndecan and glypican have been shown to have critical biological consequences [1,2,25,26,27,28].

PTN is known to bind sulfated GAG, including CS, dermatan sulfate (DS), heparan sulfate (HS), and heparin [29,30,31]. CS contains mostly the disaccharide unit composed of β1-4-linked N-acetylgalactosamine (GalNAc) and glucuronate (GlcA). GalNAc in CS can be 4-O-sulfated or 6-O-sulfated, with a small percentage of GalNAc sulfated at both positions. HS and heparin are mostly composed of disaccharide units containing α1-4-linked glucosamine (GlcN) and iduronate (IdoA). Sulfation density in heparin is significantly higher than other GAGs. GlcN in heparin can be N-sulfated at position 2 as well as O-sulfated at positions 6 and 3. IdoA is O-sulfated at position 2. The tri-sulfated disaccharide of N-sulfoglucosamine 6-O-sulfate (GlcNS6S) and Iduronate 2-O-sulfate (IdoA2S) is one of the most common disaccharides found in heparin.

Due to the importance of PTN–GAG interactions, the mechanism controlling PTN’s GAG specificity is crucial for determining the biological activities of PTN. PTN is a highly basic protein composed of two thrombospondin type 1 repeat domains flanked by termini rich in lysines [3,29,32]. Both structured domains contain a strong basic patch. Basic amino acids in the C-terminal domain (CTD) are clustered into two groups, referred to as cluster 1 (K84, R86, K107) and cluster 2 (K68, K91, R92). Arrangement of basic amino acids in the N-terminal domain (NTD) is more diffuse. The highly basic nature of PTN makes it an ideal protein for binding GAG. The location of PTN’s GAG-binding site has been investigated with both mass spectrometry (MS) and solution NMR. In particular, a lysine-protection MS study carried out by Fernig and coworkers showed that residues K49 (NTD), K54 (NTD), K60 (linker), K61 (linker), K68 (cluster 2), and K84 (cluster 1) are involved in heparin binding [33]. NMR chemical shift perturbation analysis showed that heparin hexasaccharides (dp6) elicited the most chemical shift changes in residues in the linker between CTD and NTD (residues N58 to E66), as well as residues around basic amino acid cluster 2 of CTD. Moreover, NTD and CTD bind long GAGs co-operatively, but can bind short heparin oligosaccharides in a relatively independent manner [3,34]. Despite this information on PTN–GAG interactions, high-resolution structural information on PTN–GAG interactions is sparse. Although solution NMR chemical shift perturbation studies have been extremely successful at investigating interactions of PTN with GAGs, atomic resolution information on PTN–GAG interactions has been harder to obtain, owing to both a lack of structurally homogeneous GAG oligosaccharides and difficulties in detecting intermolecular NOEs between GAG and proteins. In addition, even though PTN’s affinity for GAG largely depends on the charge density of GAG [34], whether different sulfate groups or their specific patterning contribute differently to PTN binding is also not clear.

In this study, we used solution NMR and selective isotope labeling schemes to analyze the intermolecular contacts between PTN and a heparin dp6 with the sequence ΔUA2S–GlcNS6S-IdoA2S–GlcNS6S-IdoA2S-GlcNS6S (DP6-C, Supplementary Figure S1). Previously, we have investigated the complex using traditional ^13^C-filtered/edited HSQCNOESY experiments designed to elucidate intermolecular contacts between uniformly ^13^C,^15^N-labeled PTN and unlabeled DP6-C [34]. However, such experiments suffer from low sensitivity because of the additional ^1^H transverse relaxations that take place during the isotope filtering steps, leading to fewer detectable intermolecular contacts. As a result, the data only confirmed that lysines were involved in the interactions, but unambiguous assignments were not possible due to chemical shift degeneracies among lysines and the limited resolution of the data. In this study we used two selective isotope labeling schemes and unfiltered NOESY experiments to achieve higher sensitivity in detecting intermolecular NOEs as well as lower ambiguity in their assignments. Our data showed that, in addition to the side chains of arginines and lysines, tryptophans also mediate the interactions between PTN and DP6-C. In particular, ^15^N-edited NOESYHSQC of ^2^H,^15^N-labeled PTN with DP6-C unambiguously identified residues K49, R52, W59, W74, G110, K111, and K114 as being involved in binding DP6-C. These residues are from the basic amino acid cluster 1, the C-terminus, and the linker connecting CTD and NTD. Most residues are located in the interface between the NTD and the CTD. Interestingly, the strongest NOE cross-peaks were between PTN and either IdoA2S.H2 or ΔUA2S.H3, indicating that 2-O-sulfated uronates play an important role in binding PTN. H5, H3, and H2 protons on GlcNS6S also contact amino acid side chains in PTN. In addition, 2D homonuclear NOESY of deuterated PTN with protonated lysines showed that K54 (NTD) and K68/91 (cluster 2) were also involved in binding DP6-C, and the NOE data are not consistent with a single binding orientation for DP6-C. Due to the strong contacts between uronate protons and PTN observed in the NMR data, we also tested the importance of 2-O-sulfation on PTN binding by titrating PTN with selectively desulfated heparin dp6. The data showed that 2-O-desulfation and N-desulfation/N-acetylation of heparin significantly reduced PTN’s GAG affinity, while 6-O-desulfation reduced PTN’s GAG affinity to a more modest extent. These results imply that PTN has a preference for the high-density sulfate cluster produced by the GlcNS6S-IdoA2S disaccharide. Using these results, molecular models of PTN domains in complex with DP6-C were constructed.

## 2. Materials and Methods

### 2.1. Expression and Purification of PTN WT and Truncation Mutants

The wild-type and truncated PTN (S13 to K114) were cloned into the pET-15b vector and transformed into Origami B (DE3) (Novagen) for bacterial expression. Recombinant expression and purification of PTN followed previous protocols [34]. In particular, all protein expression was achieved by growth in M9 media at 37 °C to an OD_600_ of 0.8, followed by overnight induction with 0.25 mM of IPTG at room temperature. ^15^N-labeled proteins were produced by supplementing the media with ^15^NH_4_Cl. Cell pellets were harvested in 20 mM of Tris (pH 7.5), 0.2 M NaCl buffer, and lysed through sonication after incubation in 1 mg/mL of lysozyme for 20 min at room temperature. Protein extraction from the supernatant was performed through cation-exchange chromatography with a 5 mL HiTrap SP column (Cytiva Life Sciences, Marlborough, MA, USA) in 20 mM of Tris buffer (pH 7.5) with 0.2 M NaCl. Protein was eluted using a NaCl gradient of 0.2 to 1.5 M.

### 2.2. Deuteration and Selective Protonation of PTN

Samples of ^2^H-labeled PTN were prepared by growth in deuterated M9 containing deuterated glucose. Cultures were first grown in LB media overnight starting from a single colony. The next day, 1 mL of the culture was pelleted through gentle centrifugation (5000× *g*) and resuspended in D_2_O M9. Cells were used to inoculate 25 mL of D_2_O M9 supplemented with 4 g/L of deuterated glucose and 1 g/L of ^15^NH_4_Cl, overnight. The entire D_2_O M9 starter culture was used to inoculate a 250 mL D2O M9 culture containing 4 g/L of deuterated glucose and 1 g/L of ^15^NH_4_Cl. Cultures were grown at 37 °C until an OD_600_ of 0.8, at which point the culture was induced with 0.25 mM of IPTG and the temperature was lowered to 20 °C. The induction time was ~16 h. To selectively protonate lysine, 400 mg/L of L-lysine was added 0.5 h before induction.

### 2.3. Generation of GAG Fragments

Heparin (Toronto Research Chemicals, North York, ON, Canada) was depolymerized with Heparinase I. Specifically, 1 g of heparin was digested using 0.8 IU of heparinase I in 20 mL of 50 mM NH_4_CH_3_CO_2_, 5 mM CaCl_2_, pH 7.0 buffer at 30 °C, until depolymerization was approximately 30% complete, as estimated using A_232_. Digested fragments were separated via size exclusion chromatography on a 2.5 × 175 cm Bio-Rad Biogel P10 column with a flow rate of 0.2 mL/min. Heparin dp6 were further separated using SAX HPLC with a 4 × 250 mm Waters Spherisorb SAX column to produce pure DP6-C oligosaccharides. Selectively desulfated heparin dp6 was purchased from Iduron (Alderly Edge, UK). Concentrations of all GAG samples were quantified using the carbazole assay [35].

### 2.4. Collection and Analysis of NMR Data

NMR data for PTN were collected either on a Bruker 600 MHz Avance III HD spectrometer equipped with a Prodigy probe or an Agilent 800 MHz Inova spectrometer equipped with a cold probe. PTN samples used for data collection contained 0.1–0.3 mM of PTN in 10 mM of MES and 150 mM of NaCl (pH 6.0). For samples containing DP6-C, two to three molar equivalents of DP6-C were added to each sample. F3-^13^C-filtered/F1-^13^C-edited NOESY was collected on ^13^C/^15^N-labeled PTN in the presence of DP6-C in 100% D_2_O. 2D homonuclear NOESY was collected on ^2^H-labeled PTN with ^1^H-labeled lysine in 100% D_2_O. ^15^N-edited NOESYHSQC experiments were collected on ^2^H/^15^N-PTN 90% H_2_O/10% D_2_O. All NOESY experiments used a mixing time of 0.2 s. ^15^N carrier frequency was set to either 120 ppm for amides and guanidine or 30 ppm for ε-amino groups. ^13^C carrier was set to 43 ppm. Titrations of wild-type PTN with unmodified and 2-O-desulfated heparin dp6 used samples with 0.1 mM of PTN. The titration of 6-O-desulfated heparin dp6 used 0.09 mM of wild-type PTN, and the titration of N-desulfated/N-acetylated heparin dp6 used 0.05 mM of wild-type PTN. In all titrations, heparin dp6 was added to concentrations of 0.1, 0.2, 0.3, 0.4, 0.6, 0.8, and 1.0 mM. All data processing was performed with NMRPipe [36] and analyzed using NMRView [37]. The sequence number of PTN was based on mature PTN. Normalized chemical shift changes were calculated using the equation $\sqrt{\Delta H^{2}+{\left(0.2\Delta N\right)}^{2}}$, where Δ*H* and Δ*N* are the changes in amide proton and nitrogen chemical shifts in ppm units, respectively. Dissociation constants of binding (*K_d_*) were calculated from the normalized chemical shift changes using the one-to-one binding model implemented in the software xcrvfit (http://www.bionmr.ualberta.ca/bds/software/xcrvfit/ (accessed on 23 December 2021)). In particular, the ligand-induced chemical shift data were fitted to the equation $\Delta \delta =\Delta \delta_{max}\frac{-\left(P_{t}+L_{t}+K_{d}\right)+\sqrt{{\left(P_{t}+L_{t}+K_{d}\right)}^{2}-4P_{t}L_{t}}}{2}$, where Δ*δ* is the chemical shift change, Δ*δ_max_* is the maximum chemical shift change, *P_t_* is the total protein concentration, and *L_t_* is the total ligand concentration.

### 2.5. Computational Modeling of the PTN-Heparin Complex

The molecular dynamics simulation package AMBER [38] was used to create models of the PTN domains bound to DP6-C. The solution structure of PTN was used as the starting model. DP6-C was constructed using parameters in the GLYCAM06 parameter set [39]. Both IdoA2S were in the ^1^C_4_ conformation and ΔUA2S was in the default conformation specified by GLYCAM06. The sulfate groups were added using the sulfate geometry specified in GLYCAM06 parameters and charges of the sulfate atoms were derived from ab initio calculation based on methyl sulfates [40]. DP6-C was docked onto PTN domains by first placing the ligand about 30 Å away from the domain’s GAG-binding face at different orientations. A one-nanosecond implicit solvent molecular dynamics simulation of the system was first carried out using ambiguous NMR distance constraints derived from NOESY data (Supplementary Table S1 and DP6-C dihedral angles were set to the values specified by Clerc et al. [41]. Specifically, the glycosidic φ/ψ angles of the disaccharide GlcNS6S-IdoA2S were set to 80°/−170°, while the glycosidic φ/ψ angles of the disaccharide IdoA2S-GlcNS6S were set to −80°/−140°. Distance constraint energies were calculated using the r^−6^ averaging weighting scheme for ambiguous distance constraints. In stage two, a 2 ns simulation was completed with all atoms free and only the DP6-C dihedral angle constraints being active. To gauge the agreement between experimental data and the models, distances between PTN residues confirmed to contact DP6-C, including W18.Hε1, W20. Hε1, R52.Hε, K45.Hζ, K54.Hζ, W59.Hε1, W74. Hε1, K68.Hζ, K91.Hζ, and K111.HN, and the nearest IdoA2S.H2/ΔUA2S.H3, GlcNS6S.H3, or GlcNS6S.H5 protons in DP6-C, were calculated. The average of all distances between residues known to contact IdoA2S.H2/ΔUA2S.H3 and the IdoA2S.H2/ΔUA2S.H3 protons nearest to them was used as a measure of whether the model is consistent with the NOESY data. Similar scores were used to evaluate the agreement between NOE contacts to GlcNS6S.H3/H5 protons (Supplementary Tables S2 and S3). The binding energies were calculated using the MMPBSA module of AMBER.

## 3. Results

### 3.1. Stoichiometry of PTN Interactions with Heprin dp6

**Figure 1:**
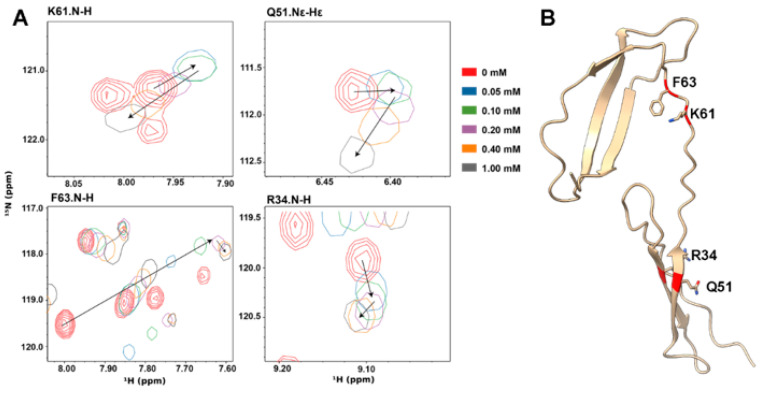
Heparin dp6 induces nonlinear chemical shift migrations in ^1^H,^15^N-labeled PTN NMR signals. (**A**) PTN-induced changes in the ^15^N-HSQC signals of PTN residues R34, Q51, K61, and F63. ^15^N-HSQC contours are colored by concentrations of heparin dp6. (**B**) Ribbon representation of PTN with side chains of R34, Q51, K61, and F63 in the stick form.

The interactions between PTN and GAG have been investigated using solution NMR in several studies [3,29,32,34]. Previous investigations showed that removing the unstructured N- and C-termini does not change the GAG affinity of PTN [3,34]. As a result, we chose to focus on GAG’s interactions with PTN’s structured domains (residues S13 to K114) in the current study. One important question that must be answered before accurate structural models of the PTN-GAG complex can be constructed is the stoichiometry of binding. Previous studies indicate that PTN’s CTD and NTD bind short GAG oligosaccharides with different affinities [32,34]. However, it is unclear whether the binding stoichiometry should be one oligosaccharide per protein or two oligosaccharides per protein. In the current study, analysis of the titration of the truncated form of PTN revealed that some residues experience non-linear chemical shift migrations. Four examples are shown in Figure 1. In these data, signals from the backbone amides of R34, K61, K63, and the side-chain amide of Q51 changed directions after the PTN-to-heparin dp6 ratio reached approximately 1:1 (Figure 1). This is a possible sign that two consecutive binding events have happened. Since CTD’s GAG affinity is considerably higher than that of the NTD [3,32,34], we think heparin dp6 most likely binds CTD first, followed by binding to the NTD. This implies that CTD and NTD must bind heparin dp6 independently, which is consistent with the fact that separation of the domains did not change each domain’s affinity for heparin dp6 [34]. Similar nonlinear chemical shift migrations were also observed in R34, Q51, and F63 of wild-type PTN (Supplementary Figure S2). Due to severe heparin-induced signal broadening, the migration of backbone amide signal of K61 in wild-type PTN could not be tracked.

### 3.2. Intermolecular NOEs between PTN and DP6-C

**Figure 2:**
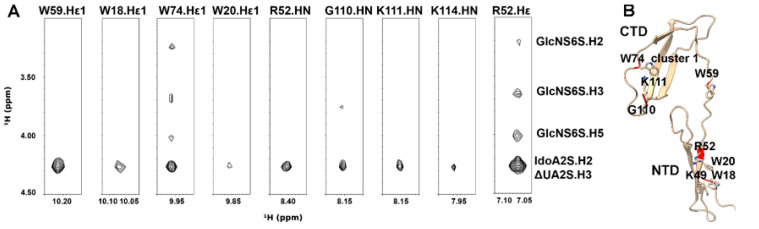
Intermolecular NOE between PTN and DP6-C seen in the ^15^N-edited NOESYHSQC of 0.3 mM ^1^H back-exchanged ^2^H,^15^N-labeled PTN with 0.9 mM DP6-C. (**A**) Strips from the NOESYHSQC spectrum showing intermolecular contacts between PTN and DP6-C. (**B**) Ribbon representation of PTN with the residues involved in intermolecular contacts colored in red.

**Figure 3:**
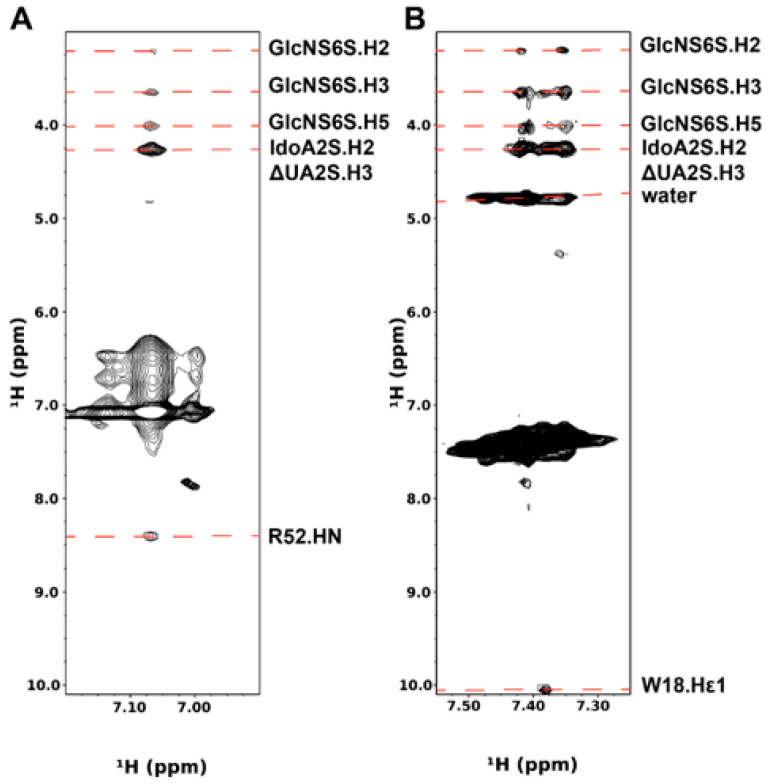
^15^N-edited NOESYHSQC of Arg and Lys side chains. (**A**) Strip of ^15^N-edited NOESYHSQC of R52.Hε. (**B**) Strip of ^15^N-edited NOESYHSQC of Lysine Hζ.

To investigate the interactions between PTN and heparin, we chose the highly sulfated heparin oligosaccharide ΔUA2S-GlcNS6S-IdoA2S-GlcNS6S-IdoA2S-GlcNS6S (DP6-C) as the ligand. Our previous study has shown that the oligosaccharide interacts with PTN with high affinity, and paramagnetically tagged versions of DP6-C produced significantly stronger paramagnetic effects in PTN than oligosaccharides with lower sulfation densities [34]. Due to the insensitivity and low resolution of the 3D ^13^C-filtered/edited HSQCNOESY experiments, we prepared ^2^H,^15^N-labeled PTN to identify intermolecular contacts to labile protons from guanidines, amides, and amines using ^15^N-edited NOESYHSQC. Besides reducing spectral complexity, ^2^H labeling also eliminated the need for isotope filters, thereby reducing relaxation and increasing signal sensitivity. Figure 2 shows the intermolecular contacts identified in the ^15^N-edited NOESYHSQC of ^2^H/^15^N-labeled PTN with DP6-C. The identity of these cross-peaks as intermolecular NOEs was further confirmed by their absence in the ^15^N-edited NOESYHSQC of ^2^H/^15^N-labeled PTN alone. The most intense contacts between PTN and DP6-C involve arginine, lysines, and tryptophans. Although there are five arginines in PTN, only the side chains of R52 and R92 can be observed in the ^15^N-edited NOESYHSQC. The other three arginines are near tryptophans. Their Hε signals underwent significant broadening in the presence of DP6-C, possibly due to dynamic interactions introduced by the competition for the arginine side chains between cation–pi interactions with tryptophans and electrostatic interactions with heparin dp6. Furthermore, even though the Hε chemical shifts of R52 and R92 are similar, we think the indicated arginine signal corresponds to R52. This conclusion is based on the observation that the Hε involved in the intermolecular contacts with DP6-C also has a cross-peak to the backbone amide proton of R52 (Figure 3A). In addition, the backbone amide of R52, but not R92, also contacts DP6-C (Figure 2). Besides R52, side-chain indole protons of tryptophans showed strong interactions with oligosaccharides as well. This was especially evident for the indole protons of W74 and W59, both of which are in the CTD. Backbone amide hydrogens of C-terminal residues G110, K111, and K114 also have contacts with the oligosaccharides.

Lysines are another group of basic amino acids that often play important roles in protein–GAG interactions. Since signals from ε-amino groups of lysines are usually not detectable because of their fast solvent exchange rates, previous studies have taken the approach of lysine methylation or cross-linking to probe interactions of lysines with proteins [42,43]. Surprisingly, lysine ε-amino signals of PTN were visible in ^15^N-edited NOESYHSQC in the presence of DP6-C (Figure 3B). This is most likely caused by interactions with DP6-C that may have slowed the solvent exchange rates of these groups. In addition, some of the side-chain amines have strong contacts with the oligosaccharide. Due to degeneracies in the chemical shifts of lysine Nζ/Hζ, unambiguous assignments of these signals were not possible. However, one of the lysines contacting DP6-C has an NOE contact to W18.Hε1. Since the only lysine near W18 is K49, we think K49 is involved in binding DP6-C.

One notable feature of the ^15^N-edited NOESYHSQC data is that the same set of DP6-C resonances were seen in all intermolecular contacts. In particular, a DP6-C resonance at 4.27 ppm appears to strongly contact all PTN protons involved in intermolecular contacts (Figure 2A and Figure 3B). Based on the known chemical shift assignments of DP6-C, this resonance corresponds to ΔUA.H3, IdoA2S.H2, and one of the methylene GlcNS6S.H6 protons (Supplementary Figure S3). However, since it is unlikely that PTN would have NOE contact with one of the methylene protons without interacting with the other one, the main contributors of the cross-peak are most likely contacts between PTN and uronate protons. Besides this signal, several amino acids also contact DP6-C resonance at 4.01 ppm, corresponding to GlcNS6S.H5. In addition, W74, R52, and the lysines contact DP6-C resonances at 3.64 and 3.21 ppm. These signals correspond to GlcNS6S.H3 and GlcNS6S.H2 (Supplementary Figure S3).

**Figure 4:**
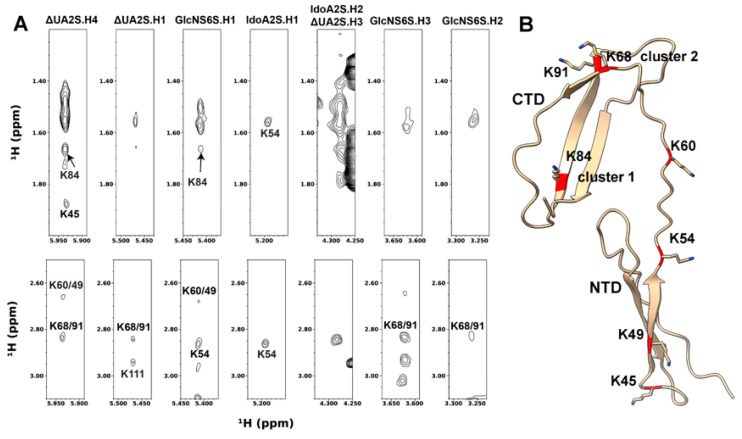
Intermolecular NOE between PTN and DP6-C seen in the 2D homonuclear NOESY of 0.2 mM ^2^H-labeled PTN with ^1^H-labeled lysines in the presence of 0.9 mM DP6-C. (**A**) Strips from the 2D-NOESY spectrum showing intermolecular contacts between PTN and DP6-C. (**B**) Ribbon representation of PTN with the residues involved in intermolecular contacts colored in red.

Since ^15^N-edited NOESYHSQC spectra only provide intermolecular NOE between DP6-C and labile protons, we also prepared deuterated PTN containing ^1^H-labeled lysines and acquired 2D homonuclear NOESY of these samples in the presence and absence of DP6-C. As a result of the selective protonation, no filtering was necessary to obtain intermolecular contacts between PTN’s lysines and DP6-C. We chose lysines for selective protonation because they are crucial in binding DP6-C, yet very little is known about the role of each lysine in binding the oligosaccharide. In the absence of DP6-C, all lysines are visible in the Hα region of the spectrum and can be unambiguously assigned (Supplementary Figure S4). The addition of DP6-C led to significant chemical shift changes. Signal intensities of K54 and K68 also decreased greatly. Fortunately, most signals in the Hα region were still visible and can be assigned (Supplementary Figure S4). This allowed us to obtain most of the proton assignments for lysine side chains (Supplementary Table S4). Even though signal overlaps between resonances in PTN and DP6-C as well as chemical shift degeneracies among lysines prevented all the intermolecular NOE cross-peaks from being identified, the high resolution and sensitivity of the spectrum mean a considerable amount of information was still available in the data. In agreement with the ^15^N-edited NOESYHSQC data, IdoA2S.H2, GlcNS6S.H2, and GlcNS6S.H3 showed intermolecular NOEs with PTN lysines. Additionally, all four anomeric protons and ΔUA.H4 also have intermolecular NOE cross-peaks in the spectrum. The high resolution of 2D NOESY allowed accurate identification of many intermolecular NOE cross-peaks. Figure 4 shows the intermolecular NOE identified in the 2D NOESY. In the case of ΔUA2S.H4, intermolecular NOE cross-peaks to K45 (NTD) and K84 (cluster 1) can be unambiguously assigned. A resonance corresponding to Hε of K68 and K91 in cluster 2 can also be seen. Since these lysines are located at different parts of the protein, these contacts can only be explained by the existence of two or more binding modes that place ΔUA2S.H4 in different locations on the protein. Besides these contacts, IdoA2S.H1 makes contact mainly with K54 (NTD), protons from K54 and K84 match the intermolecular NOE cross-peaks seen with GlcNS6S.H1, and the Hε chemical shifts of K68/91 (cluster 2) and K111 (C-terminus) match the intermolecular cross-peaks in the strip of ΔUA2S.H1.

Lastly, the F1-^13^C-edited, F3-^13^C-filtered HSQCNOESY spectrum of fully ^13^C/^15^N-labeled truncated PTN with DP6-C was also acquired. The spectrum confirmed that lysines are involved in GAG binding (Supplementary Figure S5). In particular, intermolecular NOE cross-peaks involving ΔUA.H4, GlcNS6S.H2, and GlcNS6S.H3 were seen, and these cross-peaks were consistent with lysine side chains, especially Cγ/Hγ, Cδ/Hδ, and Cε/Hε. This is in agreement with the 2D NOESY spectrum of deuterated PTN with ^1^H-labeled lysines.

### 3.3. Sulfate Specificity of PTN

**Figure 5:**
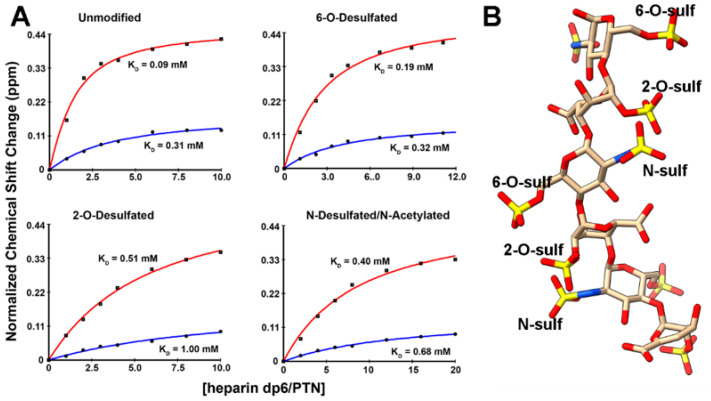
Effects of selective desulfation of heparin dp6 on its interactions with PTN. (**A**) Binding curves and apparent Kd of PTN residues T34 (blue, NTD) and N96 (red, CTD) when titrated with either unmodified or selectively desulfated heparin dp6. (**B**) Structure of DP6-C with the IdoA2S in the 1C4 conformation and glycosidic dihedral angles set to the minimal energy values.

The ^15^N-edited NOESYHSQC spectra indicate that uronate protons have the strongest intermolecular contacts with PTN. This raises the question of whether PTN has a preference for 2-O-sulfates. To investigate PTN’s specificity for sulfate positions, we titrated PTN with commercially available, selectively desulfated heparin dp6. These dp6 oligosaccharides underwent either selective 6-O-desulfation, selective 2-O-desulfation, or selective N-desulfation/N-acetylation. Figure 5A shows the binding curves derived from the ^15^N-edited HSQCs taken during the titrations. All selectively desulfated oligosaccharides induced the same chemical shift migration patterns in PTN, indicating that the oligosaccharides interact with PTN similarly (Supplementary Figure S6). However, the magnitudes of chemical shift migration as well as the rates of chemical shift changes differ among the oligosaccharides. Calculations of the apparent Kd of binding showed that the unmodified heparin dp6 induced the largest chemical shift changes and had the highest apparent affinity for PTN. The 6-O-desulfation of heparin dp6 resulted in only modest affinity decreases. However, 2-O-desulfation and N-desuflation/N-acetylation both significantly decreased PTN’s apparent affinity for these oligosaccharides. These data indicate that 2-O-sulfate and N-sulfate groups play crucial roles in PTN’s interaction with GAGs.

We think that these results may be explained by the conformation of heparin. Analysis of heparin conformation in both free and protein-bound forms have shown that glycosidic dihedral angles in the disaccharide GlcNS6S-IdoA2S are such that the N-sulfate group on GlcNS6S and 2-O-sulfate on IdoA2S in the disaccharide are close to each other when IdoA2S is in either the ^1^C_4_ or ^2^S_0_ conformation [41,44,45,46] (Figure 5B). These inter-residue high-density sulfate clusters should be ideal binding sites for basic amino acids. If the trisaccharide motif GlcNS6S-IdoA2S-GlcNS6S is present, the 6-O-sulfate from the reducing end, GlcNS6S, is also not far away. However, the glycosidic dihedral angles between IdoA2S and GlcNS6S have two energy minima [41], and therefore the bond may be more flexible than the glycosidic bond in the disaccharide GlcNS6S-IdoA2S. Such flexibility may introduce additional movements that destabilize the interactions of 6-O-sulfate groups with PTN. Similarly, the rotatable bond between C5 and C6 of GlcNS6S may also introduce extra dynamics that prevent stable interactions with PTN. We think the fact that 2-O- or N-desulfation significantly diminishes PTN’s affinity for GAG is a strong indication that the GlcNS-IdoA2S disaccharide is an important binding motif for PTN.

### 3.4. Model of the PTN-GAG Complex

To investigate if the experimental data are sufficient to create high-resolution models of the PTN-DP6-C complex, we attempted the docking of DP6-C onto PTN domains. Since GAG interactions of CTD and NTD are relatively independent and not mutually exclusive, we chose to model DP6-C’s interactions with CTD and NTD separately. Based on the NOESY data, the CTD binding site should consist of W59 (linker), K68/91 (cluster 2), W74 (cluster 1), and K111 (C-terminus), while the NTD binding site should include mostly W18, W20, R52, and K54. The docking was implemented using the MD simulation software suite AMBER [38]. AMBER was chosen as the docking software because of the availability of forcefield parameters for GAG oligosaccharides [39] and the ability to define the pucker of each monosaccharide. This allowed us to create parameter files for DP6-C in which the IdoA2S residues were assigned the conformation of ^1^C_4_ and the GlcNS6S residues were assigned the conformation of ^4^C_1_. Due to the sparse nature of the intermolecular contact information, which can often lead to biased and distorted models because of overfitting, we chose to dock DP6-C onto PTN using the principle that 2-O-sulfates are likely to be the center of the interactions with the arginines, lysines, and tryptophans identified in the ^15^N-edited NOESYHSQC data. In particular, to dock DP6-C to CTD, we specified ambiguous distance constraints between the terminal oxygens in the 2-O-sulfates and the Hε1 of W74 and W59, and Hζ of K68/91 and K111. For docking to NTD, ambiguous constraints were specified between the 2-O-sulfates and Hε of R52, Hζ of K49 and K54, and Hε1 of W18 and W20. An unambiguous distance constraint between K45.Hζ and ΔUA2S.H4 was also included. The full set of ambiguous distance constraints used in the docking is shown in Supplementary Table S1. The resulting models were then evaluated based on the agreement between the experimental NOESY data and the corresponding intermolecular distances in the model (see descriptions below as well as in Section 2 for details). It should also be noted that accurate calibration of intermolecular distances is usually not possible. Comparison of the intermolecular NOE intensities with intramolecular NOE intensities indicates that the intermolecular contacts are in the weak range and should represent distances greater than 4 Å. We think this method of including the experimental information is more representative of the relevant physical contacts, resulting in structures that are more likely to be accurate representations of the actual interactions.

DP6-C was docked onto each domain using these distance constraints with a HADDOCK-like algorithm [47]. Specifically, DP6-C was placed at a distance of ~30 Å away from the protein at 64 different orientations generated by rotating DP6-C around the X-, Y-, and Z-axes in 90° increments. One nanosecond of implicit solvent molecular dynamics (MD) simulation was carried out to dock the oligosaccharides onto the protein using the distance constraints while holding the backbone of the structured parts of the domains fixed. The resulting structures were then refined with two nanoseconds of implicit solvent MD simulation without distance constraints. Structures from the last nanosecond of the simulation were ranked based on their agreements with NOESY data. Theoretical ΔG of binding was also calculated for each model using AMBER’s MMPBSA module. Since the domains are not expected to undergo large conformation changes after binding GAG, backbone deviation from the starting structure was also used as a criterion.

**Figure 6:**
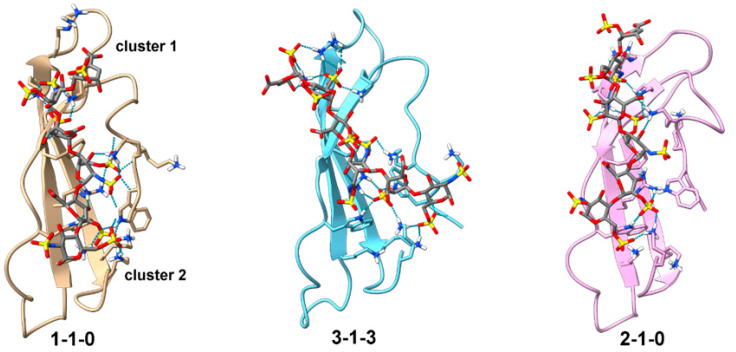
Top three models of CTD docked with DP6-C. CTD is shown in the ribbon representation. DP6-C and side chains of amino acids near DP6-C are shown in stick representations. Hydrogen bonds and electrostatic contacts between CTD and DP6-C are shown as dotted cyan lines. Models were created by docking DP6-C onto CTD using distance constraints between 2-O-sulfate groups and residues known to contact uronate protons. This was followed by simulation without distance constraints. The models were then ranked based on the calculated binding energy and distances between PTN residues and DP6-C protons known to have intermolecular contacts in the ^15^N-edited NOESYHSQC.

To rank the CTD structures, the shortest distances between W59.Hε1, W74.Hε1, K68/91.Hη, K111.HN, and the IdoA2S.H2/ΔUA2S.H3 protons nearest to each of them were calculated, and the average of these distances was used as the primary criterion for scoring the models. The binding energies and other scores of the top 10% of models with the shortest distance between IdoA2S.H2/ΔUA2S.H3 and the four residues are shown in Supplementary Table S2. Out of these models, structures of the three models with the most favorable binding energies are shown in Figure 6 and their PDB coordinates are included in Supplementary File S1. These structures all exhibited similar binding modes. Specifically, the oligosaccharide straddles the basic face of CTD, cross-linking basic amino acids from cluster 1 and cluster 2. In all three, the reducing end of DP6-C is close to cluster 1, while the non-reducing end is close to cluster 2. The linker is also pulled towards CTD compared to the starting structure. DP6-C in these models interacted with CTD in slightly different ways. In both models 1-1-0 and 2-1-0, the non-reducing end ΔUA2S does not have significant interactions with the CTD, while all six monosaccharides in model 3-1-3 have significant interactions with the CTD. In addition, DP6-C in models 1-1-0 and 2-1-0 is positioned in such a way so as to allow the reducing end tri-sulfate cluster to interact with cluster 1 and the linker, while the non-reducing end tri-sulfate cluster interacts with cluster 2. The DP6-C in model 3-1-3, however, is positioned to allow only the reducing end tri-sulfate cluster to bind cluster 1 and the linker. The non-reducing end tri-sulfate cluster in model 3-1-3 is not in a position to interact with cluster 2. Instead, the disulfate cluster formed by ΔUA2S and the 6-O-sulfate from the neighboring GlcNS6S engaged with cluster 2. Since atoms in ΔUA2S had intermolecular NOE cross-peaks with lysines in PTN and the conformation of IdoA2S residues in models 1-1-0 and 2-1-0 deviate more from the ideal ^1^C_4_ shape than in model 3-1-3, we think model 3-1-3 is in better agreement with the experimental data. For all the models, the average distance from DP6-C-contacting residues identified in ^15^N-edited NOESYHSQC to IdoA2S.H2/ΔUA2S.H3 was considerably less than distances to GlcNS6S.H5/H3. This is also in agreement with the NOE cross-peak intensities observed in the ^15^N-edited NOESYHSQC.

**Figure 7:**
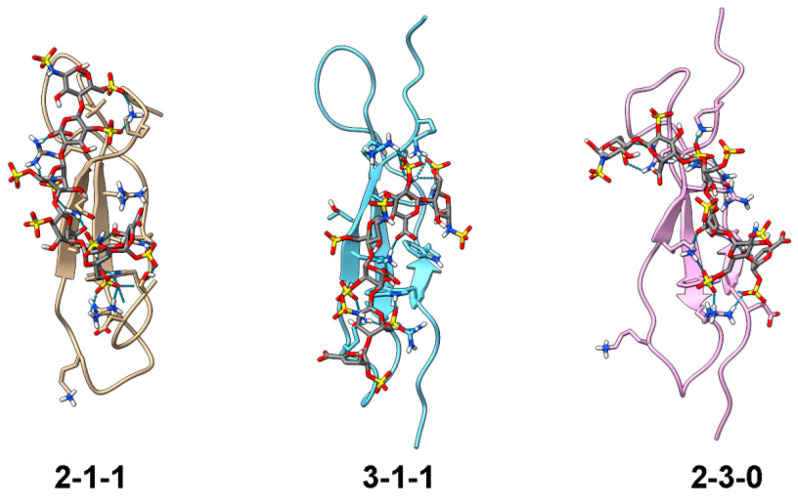
Top three models of NTD docked with DP6-C. NTD is shown in the ribbon representation. DP6-C and side chains of amino acids near DP6-C are shown in stick representations. Hydrogen bonds and electrostatic contacts between CTD and DP6-C are shown as dotted lines. Models were created by docking DP6-C onto NTD using distance constraints between 2-O-sulfate groups and residues known to contact uronate protons. This was followed by an MD simulation without distance constraints. The models were then ranked based on the calculated binding energy and distances between PTN residues and DP6-C protons known to have intermolecular contacts in the ^15^N-edited NOESYHSQC.

For docked NTD structures, the shortest distances between W18.Hε1, W20.Hε1, R52.Hε, K54.Hζ, and the IdoA2S.H2/ΔUA2S.H3 protons nearest to each of them were calculated and included in the primary ranking criterion. The score also included the distance between K45.Hζ and ΔUA2S.H4, which was one of the unambiguous contacts identified in the NOESY data. The binding energies and other scores of the top 10% of models with the shortest distance between these five residues and IdoA2S.H2/ΔUA2S.H3/ΔUA2S.H4 are shown in Supplementary Table S3 and structures of the three models with the most favorable binding energy are shown in Figure 7. The PDB coordinates of the models are included in Supplementary File S1. Overall, the predicted binding energies of DP6-C to NTD are significantly less favorable compared to the DP6-C binding energies of CTD, which is in qualitative agreement with the experimental GAG affinities of the domains. In all three models, the reducing end tri-sulfate cluster of DP6-C interacts with the basic amino cluster formed by R35, R52, and K54, while the disulfate cluster of 6-O-sulfate and 2-O-sulfate at the non-reducing end interacted with R39 and K49. Even though a distance constraint between K45 and ΔUA2S.H4 was included in the modeling, only the K45 in model 3-1-1 is in a position to interact with DP6-C. This may be a consequence of the instability of K45′s interactions with DP6-C and the dominant role of the basic cluster around R53/K54 of NTD in binding DP6-C.

## 4. Discussion

In this study, we used solution NMR to investigate the interactions of PTN with a structurally defined heparin dp6 at atomic resolution. As far as we know, this study is the first attempt at using unfiltered NOESY and deuteration with selective protonation to study PTN–GAG interactions. This approach yielded a significant amount of valuable information. In particular, PTN residues K49, R52, K54, W59, W74, K84, K68/91, G110, K111, and K114 were shown to have significant interactions with DP6-C. Most of the DP6-C-binding lysines identified here were also shown to be involved in heparin binding by the MS study of Ori et al. [33], but this is the first time that R52 and tryptophan residues were identified as heparin-contacting residues. It is worth noting that K45 (NTD), K84 (cluster 1), and K68/91 (cluster 2) all contacted the DP6-C proton ΔUA2S.H4. These lysines are distributed across different parts of PTN, thus no single binding conformation of the oligosaccharide can produce all three contacts. This implies that DP6-C has multiple binding modes and is consistent with previous studies that showed that DP6-C paramagnetically tagged at the reducing end can induce signal broadening in residues throughout PTN [34].

Results in this report also provided new insights into the sulfation pattern preferences of PTN. In particular, the strong intermolecular NOEs between uronate protons and PTN seen in ^15^N-edited NOESYHSQC prompted us to investigate the role of 2-O-sulfation in PTN’s interaction with heparin. Titrations of PTN with selectively desulfated heparin dp6 showed that 2-O-desulfation and N-desulfation/N-acetylation decreased PTN’s affinity for heparin dp6 significantly more than 6-O-desulfation. We think that this is the result of PTN’s preference for the GlcNS6S-IdoA2S disaccharide unit. This preference most likely reflects the fact that protein–GAG interactions are more likely to be stable if the interactions are between clusters of basic amino acids on the protein and high-density sulfate clusters on the GAG. The GlcNS6S-IdoA2S disaccharide can form such a high-density sulfate cluster because the glycosidic dihedral angles preferred by the disaccharide and the ^1^C_4_ conformation of IdoA lead to the N-sulfate group from GlcNS6S being placed near the 2-O-sulfate of IdoA2S. These sulfate clusters are found periodically on both sides of the polysaccharide and are ideally positioned to have electrostatic interactions with basic amino acid clusters in PTN. The extension of the disaccharide motif to the GlcNS6S-IdoA2S-GlcNS6S trisaccharide motif also extends the sulfate cluster to a tri-sulfate cluster made up of an N-sulfate, a 2-O-sulfate, and a 6-O-sulfate. The 2-O-sulfate is at the center of the sulfate cluster and plays the crucial role of bridging the gap between the sulfate groups on the GlcNS6S. However, it is possible that the flexibility in the IdoA2S-GlcNS6S glycosidic bond and the extra degree of freedom provided by the rotation of the C5-C6 bond may be destabilizing to the electrostatic interactions, thereby reducing 6-O-sulfate’s contribution to PTN binding. It was a surprise to see that the NOE contacts seen in the ^15^N-edited NOESYHSQC are similar across all residues. This may reflect the repetitive structure of DP6-C, which is made up of two nearly identical tri-sulfate clusters, but it may also be the consequence of PTN’s specificity for a particular sulfation motif. It should also be noted that previous heparin-induced chemical shift perturbation of PTN showed that the largest chemical shift perturbations are found in residues in the linker and basic amino acid cluster 2 in CTD. However, NOE analysis in this study revealed that residues in NTD, basic amino acid cluster 1, as well as the C-terminus have the strongest interactions with DP6-C. This shows that NOE information offers a valuable complement to chemical perturbation analysis. It is also consistent with the paramagnetic relaxation enhancement (PRE) study we carried out previously on PTN [34]. In particular, paramagnetically labeled heparin dp6 produced large perturbation on residues near R52/K54 in the NTD even though the same region showed only modest heparin-induced chemical shift perturbations. The current NOESY data, however, have the advantage of being collected on PTN using a native heparin oligosaccharide rather than a chemically modified heparin oligosaccharide used in the paramagnetic study.

This study only explored the specificity of PTN for commonly occurring sulfate modifications in heparin. Our previous study measured PTN’s affinity for a heparin dp6 with a 3-O-sulfated GlcNS6S3S at its reducing end and found that PTN’s affinity for the oligosaccharide is similar to DP6-C, but the oligosaccharide produced a less paramagnetic effect than DP6-C [34]. We do not think that this necessarily implies that PTN does not prefer tri-sulfated residues such as GlcNS6S3S. Since proteins’ sulfation specificity is often context-dependent, the presence of a single GlcNS6S3S residue among residues of low sulfation density may not constitute a high-affinity ligand for PTN. Based on the fact that PTN seeks out clusters of high-density sulfates, we think that the addition of a 3-O-sulfate group to the GlcNS6S-IdoA2S disaccharide should produce a very high-density sulfate cluster that can interact with PTN with even higher affinity.

Results presented here also provided additional evidence that the domains of PTN bind heparin dp6 separately. Previous studies showed that separation of domains had little effect on the binding of heparin dp6 to each domain, but it was not clear whether CTD and NTD can bind short oligosaccharides simultaneously or exclusively [34]. In this study, we noticed that many PTN residues, most prominently K61 and Q51, exhibited significant non-linear chemical shift migration paths when titrated with heparin dp6. Our interpretation is that this is caused by the binding of heparin dp6 molecules to CTD and NTD separately, and implies that the domains may bind short oligosaccharides, such as heparin dp6, independently. The fact that residues that exhibited non-linear chemical shift migration paths were more likely to be in the linker or in regions of NTD that are close to the linker means that these residues may be sensitive to ligand binding in both domains. Since CTD’s affinity for heparin dp6 is higher, it is likely to be saturated with heparin dp6 at lower concentrations. The initial chemical shift changes of these residues may be determined by the binding of heparin dp6 to the CTD. As the heparin dp6 concentration increases and NTD becomes more saturated with heparin dp6, the chemical shift migration directions of the residues between the domains may change as a result. Although such a domain-independent binding model does not seem to agree with previous isothermal titration calorimetry (ITC) results, which indicate that the stoichiometry of binding is approximately one-to-one, it is possible that ITC only detects the binding of GAG to the high-affinity site (CTD), not the low-affinity site (NTD). This is possible because electrostatic interactions often do not produce large changes in enthalpy [48,49], making their detection difficult using ITC. It should also be noted that the existence of a minor population PTN in which the two domains co-operatively bind the same heparin dp6 molecule may exist. The linker between the domains is flexible, and given the fact that the tri-sulfate clusters on heparin are distributed on opposite sides of the molecule, it is easy to envision a scenario in which the formation of a turn at the base of the linker may allow NTD to be placed side-by-side with the CTD and in a position to interact with tri-sulfate clusters on heparin that are not interacting with CTD. This scenario is especially likely when the stoichiometry between heparin dp6 and PTN is one or less.

The fact that the domains can bind heparin dp6 independently raises the interesting question of whether PTN is capable of cross-linking proteoglycans in vivo. Oligomerization of receptors such as PTPRZ and syndecan is known to alter the activity of the receptors [25,50,51]. PTN-induced proteoglycan oligomerization may be an important mechanism in its activities. However, evidence also indicates that PTN domains most likely bind long GAG chains co-operatively since the separation of domains reduces PTN’s affinity for long GAG chains considerably [3]. This means the chance that PTN domains bind long GAG chains separately is probably low. We have previously modeled the interaction of PTN with a heparin dp12, which is long enough to span both domains [34]. Since the binding sites identified in this study are close to being contiguous, a longer GAG containing at least 12 monosaccharides can easily span all sites in both domains. This would explain why the domains bind long GAGs co-operatively.

## Supplementary Materials

The following supporting information can be downloaded at: https://www.mdpi.com/article/10.3390/biom12010050/s1, Figure S1: Structure of DP6-C, Figure S2: Non-linear chemical shift migration in wild-type PTN, Figure S3: Natural abundance ^13^C-edited HSQC of DP6-C, Figure S4: DP6-C-induced chemical shift changes in the lysines of PTN, Figure S5: Intermolecular contacts seen in the F1-^13^C-edited/F3-^13^C-filtered HSQCNOESY of PTN and DP6-C, Figure S6: Titrations of PTN with unmodified or selectively desulfated heparin dp6, Figure S7: Intermolecular distances in models of CTD and NTD bound to DP6-C, Table S1: Ambiguous constraints used to model the interactions of PTN domains with DP6-C, Table S2: Scores of the top 10% of the CTD-DP6-C models with the closest distance to IdoA2S.H2/ΔUA2S.H3, Table S3: Scores of the top 10% of the NTD-DP6-C models with the closest distance to IdoA2S.H2/ΔUA2S.H3, Table S4: Complete assignment of lysine side chains of PTN with DP6-C, File S1: PDB coordinates of the models of CTD and NTD bound to DP6-C.
