# Supplementary material for: Interactions of Pleiotrophin with a Structurally Defined Heparin Hexasaccharide
Source: Biomolecules. 2021 Dec 30;12(1):50. doi: 10.3390/biom12010050 (PMC8773689; doi:10.3390/biom12010050)
Supplement: Supplementary file 1 [file biomolecules-12-00050-s001.zip › SupplementalV2.1-Eathen.pdf]

Interaction of Pleiotrophin with a Structurally Defined Heparin Hexasaccharide

Eathen O. Ryan, Zhoumai Jiang, Hoa Nguyen, and Xu Wang

The School of Molecular Sciences, Arizona State University, Tempe, Arizona, U. S. A.

Supplemental Figures and Tables

Figure S1: Structure of DP6-C,
Figure S2: Non-linear chemical shift migrations in wild type PTN,
Figure S3: Natural abundance ^{13}C-edited HSQC of DP6-C,
Figure S4: DP6-C-induced chemical shift changes in the lysines of PTN,
Figure S5: Intermolecular contacts seen in the F1-^{13}C-edited/F3-^{13}C-filtered HSQCNOESY of PTN and DP6-C,
Figure S6: Titrations of PTN with unmodified or selectively desulfated heparin dp6,
Figure S7: Intermolecular distances in models of CTD and NTD bound to DP6-C,

Table S1: Ambiguous constraints used to model the interactions of PTN domains with DP6-C,
Table S2: Scores of the top 10 % of the CTD-DP6-C models with the closest distance to IdoA2S.H2/ΔUA2S.H3,
Table S3: Scores of the top 10 % of the NTD-DP6-C models with the closest distance to IdoA2S.H2/ΔUA2S.H3.
Table S4: Complete assignments of lysine side chains of PTN with DP6-C,

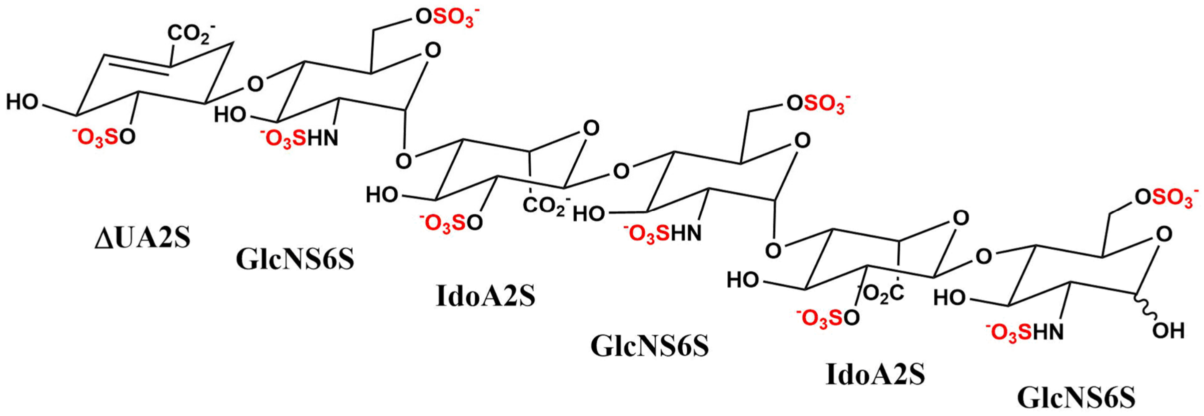

Figure S1. The structure of DP6-C

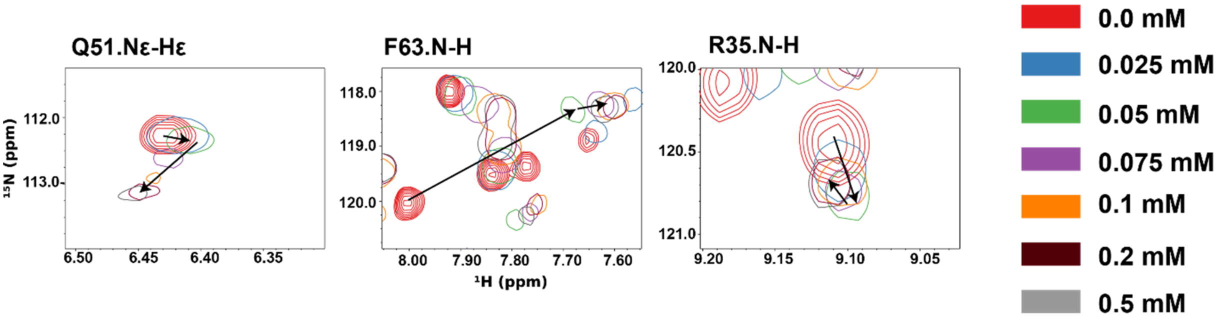

Figure S2. Non-linear chemical shift migrations of residues R35,Q51, and F63 signals seen in the titration of 0.05 mM wild type PTN by heparin dp6.

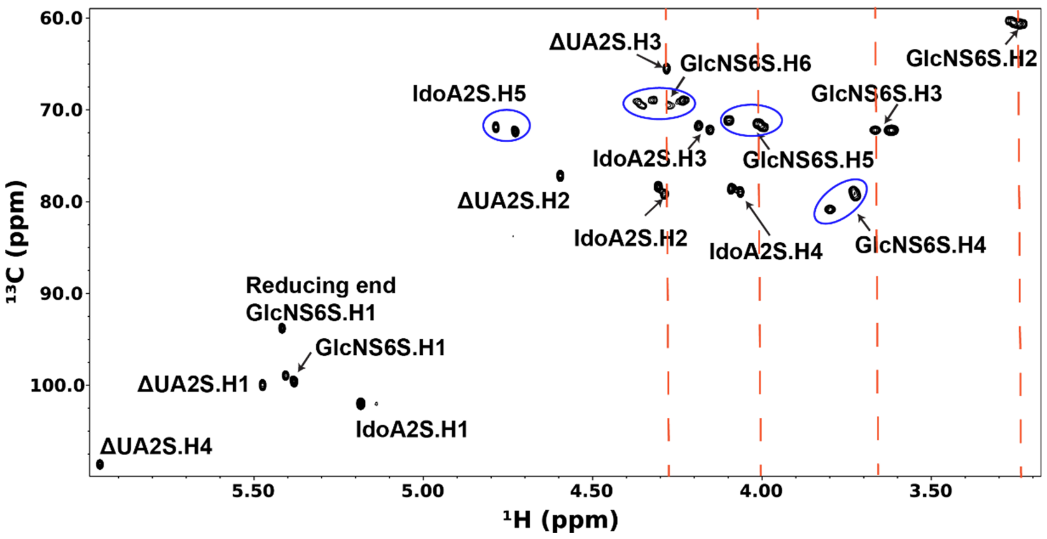

Figure S3. Natural abundance ^{13}C-edited HSQC of DP6-C. ^{1}H chemical shifts corresponding to the chemical shifts of DP6-C protons seen in ^{15}N-edited NOESYHSQC are marked with red dashed lines.

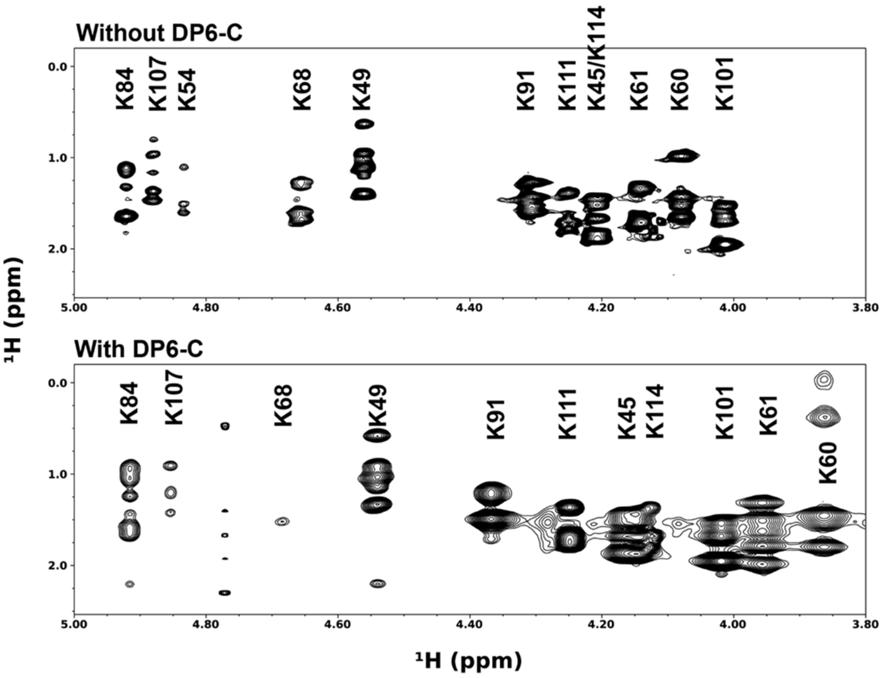

Figure S4. DP6-C-induced chemical shift changes in the lysines of PTN. Hα region of 2D homonuclear NOESY of deuterated PTN with protonated lysines in the presence and absence of DP6-C.

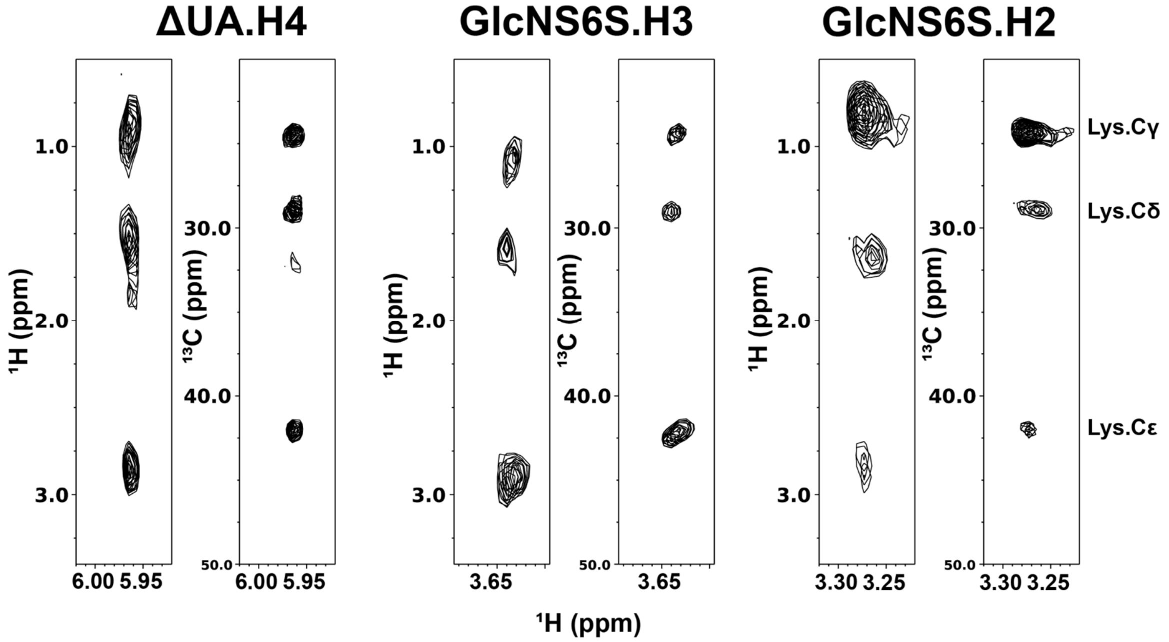

Figure S5. Intermolecular contacts seen in the F1-^{13}C-edited/F3-^{13}C-filtered HSQCNOESY of 0.3 mM ^{13}C,^{15}N-labeled PTN and 0.9 mM DP6-C.

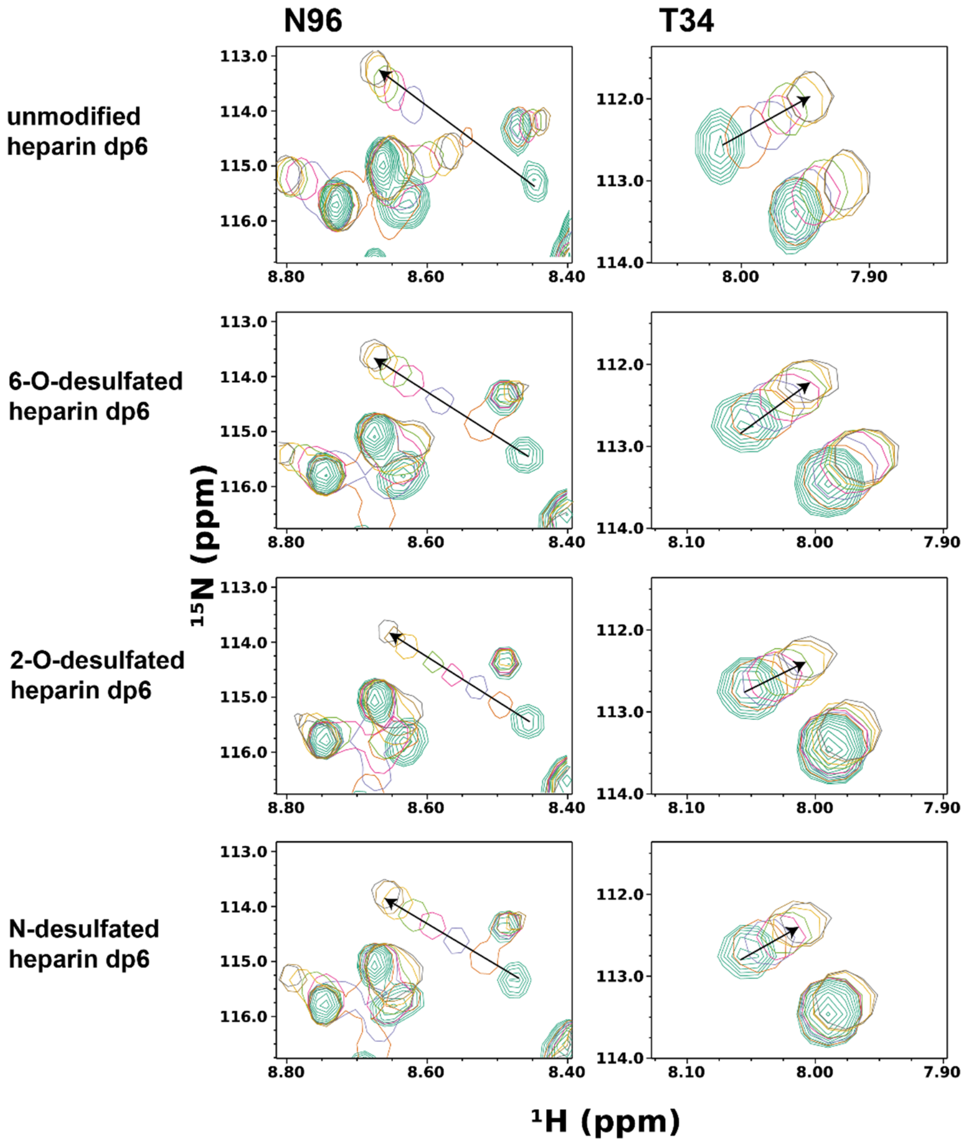

Figure S6. Migration paths of residues T34 and N96 in titrations of wild type PTN with unmodified or selectively desulfated heparin dp6. ^{15}N-edited HSQC of PTN in the absence of heparin dp6 are shown as solid green contours. Other HSQCs are shown as hollow contours.

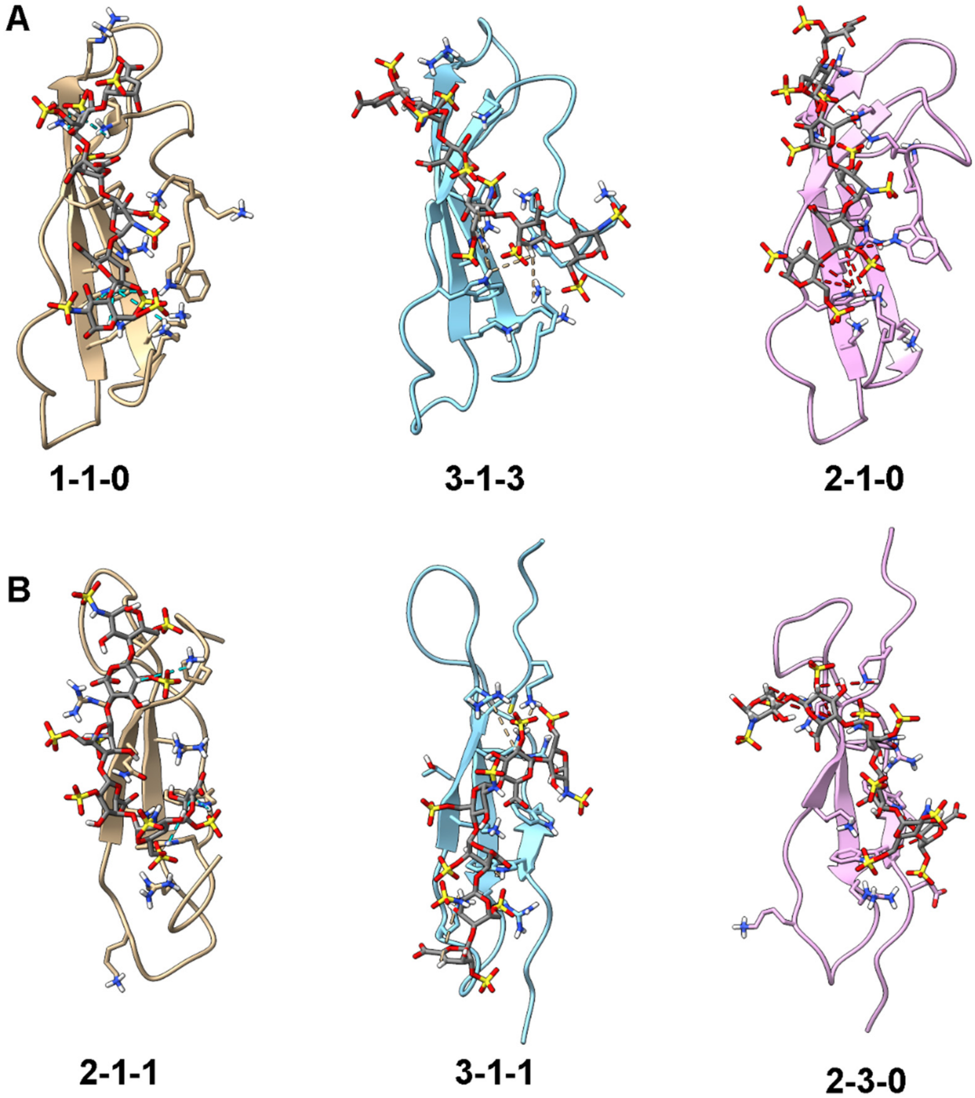

Figure S7. Intermolecular distances in models of CTD and NTD bound to DP6-C. A) The top three models of CTD bound to DP6-C. Intermolecular distances corresponding to the NOE cross peaks observed in ^{15}N-edited NOESYHSQC are shown in dashed lines. Specifically, lines are drawn between W54.Hε1, K68/91.Hζ,W74.Hε1,K111.Hζ and nearest IdoA2S.H2/ΔUA2S.H3. Connections between W54.Hε1and nearest GlcNS6S.H3/H5 are also shown. B) the top three models of NTD bound to DP6-C. Intermolecular distances corresponding to the NOE cross peaks observed in ^{15}N-edited NOESYHSQC are shown in dashed lines. Specifically, lines are drawn between W18.Hε1, W20.Hε1, R52.Hε, K54.Hζ and nearest IdoA2S.H2/ΔUA2S.H3. Connections between R52.Hη and nearest GlcNS6S.H3/H5 and K45.Hζ and ΔUA2S.H4 are also shown. To improve the visibility, the lines are colored cyan, tan, and red for models on the left, middle, and right, respectively. PDB coordinates of the models can be found in the supplementary materials.

Table S1. Ambiguous constraints used to model the interactions of PTN domains with DP6-C.

PTN domain	PTN atom	DP6-C atom	Distance range (Å)
NTD	W18.Hε1	Terminal oxygens of 2-O-sulfates	2 - 3
	W20.Hε1	Terminal oxygens of 2-O-sulfates	2 - 3
	K45.Hζ	Terminal oxygens of any 2-O-sulfates	2 - 3
	R52.Hη	Terminal oxygens of 2-O-sulfates	2 - 3
	K54.Hζ	Terminal oxygens of any 2-O-sulfates	2 - 3
CTD	W59.Hε1	Terminal oxygens of any 2-O-sulfates	2 - 3
	K68/91.Hζ	Terminal oxygens of any 2-O-sulfates	2 - 3
	W74.Hε1	Terminal oxygens of any 2-O-sulfates	2 - 3
	K111.Hζ	Terminal oxygens of any 2-O-sulfates	2 - 3

Table S2. Scores of the top 10 % of the CTD-DP6-C models with the closest distance to IdoA2S.H2/ΔUA2S.H3.

**Model**[1]	**$\Delta G_{binding}$**	**RMSD to starting structure**[2]	**Average Distance to**[3]		
			IdoA2S.H2/ ΔUA2S.H3	GlcNS6S.H3	GlcNS6S.H5
3-3-0	-86.5836	0.45384	4.65675	8.91225	7.97
3-1-3	-127.774	0.42305	5.241	8.44075	7.265
1-1-0	-136.165	0.428516	5.3225	8.25725	7.6155
3-3-3	-100.689	0.427318	5.816	8.55225	7.6585
2-1-2	-111.565	0.374486	5.87375	7.4365	6.887
1-3-2	-53.1541	0.504316	5.9305	11.337	10.917
2-1-0	-117.882	0.45625	6.0045	8.746	7.278

[1] Model numbers indicate the initial orientation of DP6-C. The triplet of the numbers specifies the sequential rotations of the ligand around X-Y-Z axes in 90° increments. For instance, 3-1-2, means the ligand was rotated 270° around the X-axis, 90° around the Y-axis, and 180° around the Z-axis.

[2] RMSD is calculated for backbone atoms of the β-strand residues 68 to 71, 83 to 92, and 102 to 108.

[3] Distance calculated by averaging the closest distances between the DP6-C atoms and a set of the CTD atoms whose intermolecular NOE cross peaks with DP6-C were observed in the NMR data. These atoms include W59.Hε1, W74. Hε1, K68.Hζ, K91.Hζ, and K111.HN.

Table S3. Scores of the top 10 % of the NTD-DP6-C models with the closest distance to IdoA2S.H2/ΔUA2S.H3/ΔUA2S.H4.

**Model**[1]	**$\Delta G_{binding}$**	**RMSD to starting structure**[2]	**Average Distance to**[3]		
			IdoA2S.H2/ ΔUA2S.H3/ ΔUA2S.H4[4]	GlcNS6S.H3	GlcNS6S.H5
2-1-1	-59.793	2.73	4.3018	8.20675	6.73575
3-1-1	-55.0464	1.63	4.342	6.7215	6.092
2-3-0	-49.4583	1.30	4.4734	6.695	6.82225
3-0-1	-37.1146	2.78	4.6826	7.47375	7.2935
2-3-1	-38.9709	2.57	4.9426	8.45725	9.724
1-3-2	-36.7284	1.88	5.0664	8.374	8.66775
3-2-1	-34.0156	1.48	6.0012	10.2203	9.883

[1] Model numbers indicate the initial orientation of DP6-C. The triplet of the numbers specifies the sequential rotations of the ligand around X-Y-Z axes in 90° increments. For instance, 3-1-2, means the ligand was rotated 270° around the X-axis, 90° around the Y-axis, and 180° around the Z-axis.

[2] RMSD is calculated for backbone atoms of the β-strand residues 16 to 23, 31 to 40, and 45 to 57.

[3] Distance calculated by averaging the closest distances between the DP6-C atoms and a set of the NTD atoms whose intermolecular NOE cross peaks with DP6-C were observed in the NMR data. These atoms include W18.Hε1, W20. Hε1, K45. Hζ, R52.Hε, and K54.Hζ.

[4] Average of the distances between W18.Hε1, W20. Hε1, R52.Hε, and K54.Hζ and the nearest IdoA2S.H2/ΔUA2S.H3 as well as the distance between K45. Hζ and ΔUA2S.H4

Table S4. Assignments of lysine side chains of deuterated PTN with protonated lysines in the presence of DP6-C. Assignments are in ppm units.

	Hα	Hβ	Hγ/δ	Hε
**K45**	4.15	1.87	1.69, 1.53, 1.43	3.02
**K49**	4.54	1.33	1.03, 0.57	2.68, 2.19
**K54**			1.56, 1.06	2.86
**K60**	3.86	1.79, 1.45	0.38, -0.05	2.66
**K61**	3.96	1.99, 1.78	1.63, 1.50, 1.31	2.91
**K68**	4.68			2.97, 2.82
**K84**	4.91	1.67, 1.57	1.25, 1.09, 0.99	2.2
**K91**	4.37	1.54, 1.48	1.25, 1.17	2.82, 2.77
**K101**	4.02	1.96	1.7, 1.63, 1.53	3
**K107**	4.85	1.42	1.22, 0.91	2.53, 1.97
**K111**	4.25	1.77, 1.69	1.63, 1.37	2.95
**K114**	4.12	1.80, 1.68	1.36	2.97
